# Photoluminescent Gold Nanoclusters as Sensing Probes for Uropathogenic *Escherichia coli*

**DOI:** 10.1371/journal.pone.0058064

**Published:** 2013-03-15

**Authors:** Po-Han Chan, Bhaswati Ghosh, Hong-Zheng Lai, Hwei-Ling Peng, Kwok Kong Tony Mong, Yu-Chie Chen

**Affiliations:** 1 Department of Applied Chemistry, National Chiao Tung University, Hsinchu, Taiwan; 2 Department of Biological Science and Technology, National Chiao Tung University, Hsinchu, Taiwan; US Naval Reseach Laboratory, United States of America

## Abstract

Glycan-bound nanoprobes have been demonstrated as suitable sensing probes for bacteria containing glycan binding sites. In this study, we demonstrated a facile approach for generating glycan-bound gold nanoclusters (AuNCs). The generated AuNCs were used as sensing probes for corresponding target bacteria. Mannose-capped AuNCs (AuNCs@Mann) were generated and used as the model sensors for target bacteria. A one-step synthesis approach was employed to generate AuNCs@Mann. In this approach, an aqueous solution of tetrachloroauric acid and mannoside that functionized with a thiol group (Mann-SH) was stirred at room temperature for 48 h. The mannoside functions as reducing and capping agent. The size of the generated AuNCs@Mann is 1.95±0.27 nm, whereas the AuNCs with red photoluminescence have a maximum emission wavelength of ∼630 nm (λ_excitation_ = 375 nm). The synthesis of the AuNCs@Mann was accelerated by microwave heating, which enabled the synthesis of the AuNCs@Mann to complete within 1 h. The generated AuNCs@Mann are capable of selectively binding to the urinary tract infection isolate *Escherichia coli* J96 containing the mannose binding protein FimH expressed on the type 1 pili. On the basis of the naked eye observation, the limit of detection of the sensing approach is as low as ∼2×10^6^ cells/mL.

## Introduction

Urinary tract infections are commonly caused by *Escherichia coli* [1], [2]. Conventionally, microbiological assays are employed to identify infected bacterial strains. However, these assays are tedious and require a few days for bacterial identification. Thus, shortening the time in conducting assay is helpful for medical diagnostics and treatment. Among the uropathogenic bacteria, *E. coli* is the most common cause of urinary tract infections [1]. *E. coli* expressing type 1 pili can mediate attachment to the mannose containing receptors [3], [4], [5] found in host tissues such as the bladder, resulting in cystitis. Mannose-based sensing methods [5]–[9] have been developed for the detection of *E. coli* with mannose binding receptors. For example, polymer based functional probes [3], [4], [5] have been demonstrated as effective probes for their target bacteria. Yang et al. generated a water-soluble, biodegradable, and fluorescent hyper-branched poly(amidoamine) linked with mannose groups for targeting *E. coli*. Fluorescence microscopy was used as the detection tool [5]. Carbohydrate-functionalized poly(*p*-phenylene ethynylene) (PPE) which can interact with *E. coli* by multivalent interactions was explored [6]. Hexameric supramolecular scaffold binding with mannose [7] was also demonstrated as useful sensing probes for *E. coli*, possessing mannose-specific receptor sites, using confocal microscopy as the detection method. Additionally, mannose-capped Au nanodots (Au NDs) for the detection of *E. coli* with mannose binding sites while employing fluorescence spectroscopy as the detection method had been reported by the research groups of Huang and Chang [8], [9]. Although the limit of detection shown in these studies was low, sophisticated instruments such as fluorescence microscopes or fluorescence spectrophotometers were used as detection tools. Furthermore, the generation of these nanoprobes took two days to more than one week. Thus, it should be desirable if the time required for the nanoprobe preparation can be shortened further and the requirement of sophisticated instruments can be eliminated.

Naked-eye-based detection assays have been used to rapidly screen target species using gold nanoparticles and AuNCs as sensing probes based on the visibility of their colors under room lighting and ultraviolet (UV) illumination [10], respectively. A concern regarding sensitivity may arise when the naked eye is used for detection. Nevertheless, naked-eye-based detection method is very convenient, especially for screening target species that do not require low detection limits. AuNCs are suitable sensing nanoprobes because of several advantages including ease of synthesis, ease of functionalization, high photostability, extremely small sizes, and unique photoluminescence properties [7], [8], [9], [11], [12], [13], [14], [15]. In the current study, a one-step synthesis approach for generating mannose-capped AuNCs (AuNCs@Mann) was demonstrated, while a screening method for uropathogenic bacteria containing mannose binding receptors was employed using the generated AuNCs@Mann as sensing probes and the naked eye as the detection tool.

## Materials and Methods

### Reagents and bacteria

Tetrachloroauric acid was purchased from Showa (Tokyo, Japan). Sodium dihydrogen phosphate was purchased from Mallinckrodt (St. Louis, MO, USA). Disodium hydrogen phosphate and sinapinic acid were obtained from Sigma-Aldrich (St. Louis, MO, USA). Luria-Bertani (LB) broth was purchased from Becton Dickinson (Franklin Lakes, NJ, USA). Agar was purchased from Amresco (Solon, OH, USA). Amicon ultra-4 filter (cut-off mass: 3 kDa) was obtained from Millipore (Billerica, MA, USA). The UTI isolate *E. coli* J96 with mannose binding proteins on the type 1 pili was purchased from Institute of Food Science (Hsinchu, Taiwan), while *E. coli* JM109 which lacking the type 1 pili activity was a routinely used cloning host.

### Synthesis of AuNCs@Mann at room temperature

Initially, we prepared mannose-capped AuNCs using a one-step synthesis approach, in which aqueous tetrachloroauric acid (1 mM, 1 mL) was stirred with mannoside that functionalized with a thiol function, namely 6-mercaptohexy α-mannopyranoside (Mann-SH) (3 mM, 1 mL), under nitrogen to prevent the formation of disulfide. The Mann-SH serves as the reducing and capping agent. Formation of the AuNCs@Mann took 48 h for completion. Procedure for the preparation of Mann-SH is described in File S1. After reaction, the resulting solution was centrifuged at 17,000 rpm for 30 min. The supernatant was filtered through an Amicon Ultra-4 filter under centrifugation in a Hettich centrifuge (Model: 2002-01, Tuttlingen, Germany) equipped with a rotor with the radius of 8.5 cm at 5,500 rpm for 30 min. The solution left on the filter was redissolved in 3 mL of deionized water. The mixture was filtered according to the abovementioned procedures to remove the un-reactivated species. The same rinse procedures were repeated again. The AuNCs@Mann were collected from the lyophilized resultant solution. The sensing solution of the 1.36 mg/mL of AuNC used in the current work was prepared from the lyophilized AuNCs@Mann.

### Synthesis of AuNCs@Mann under microwave heating

Mann-SH (3 mM, 2 mL) was stirred with tetrachloroacuric acid (1 mM, 2 mL) for 10 min followed by microwave-heating (power: 90 W; 5 min per heating cycle) in consecutive heating cycles in a domestic microwave oven. After seven cycles, the resulting solution was centrifuged in a Hermle centrifuge (model: Z232 K, Munich, Germany) equipped with a rotor with the radius of 7.5 cm at 17,000 rpm for 30 min. The supernatant was filtered through an Amicon Ultra-4 filter under centrifugation at 5,500 rpm for 30 min. The solution left on the filter was redissolved in 3 mL of deionized water. The mixture was filtered according to the aforementioned procedures in order to remove the unreacted species. The same rinse procedures were repeated several times and solid AuNCs@Mann were harvested from the lyophilization of the washed AuNCs@Mann solution. The sensing solution containing 1.36 mg/mL of AuNCs@Mann was prepared from the lyophilized AuNCs@Mann.

### Characterization of the AuNCs@Mann by high resolution transmission electron microscopy (HRTEM)

The AuNCs@Mann were characterized by a JEM-2100F field emission transmission electron microscope (JEOL, Japan) operating at a 200 kV accelerating voltage. The samples for HRTEM were prepared by drying sample droplets on a 200-mesh Cu grid, coated with formvar/carbon films.

#### Bacterial culture

*E. coli* J96 with mannose binding proteins and *E. coli* JM109 lacking mannose binding activity were initially cultured in Luria Broth (LB) for 24 h and then rinsed with phosphate buffered saline (PBS, pH 6.5) solution twice by centrifugation in a Thermo centrifuge (model: D-37520, Waltham, USA) equipped with a rotor with the radius of 8.0 cm at 4000 rpm for 10 min. PBS solution (pH 6.5) was prepared by mixing aqueous disodium hydrogen phosphate (20 mM) and aqueous sodium dihydrogen phosphate together. The bacterial cells were resuspended in a PBS solution to prepare a suspension with optical density (OD) at a wavelength of 600 nm (OD600) equal to 1. OD600 1 is equal to ∼1.43×109 *E. coli* cells/mL. We then took 10 µL of the bacterial solution and mixed it with a yeast solution (10 µL, 10 mg/mL) for the yeast agglutination test which could confirm whether the bacteria contain mannose binding sites or not.

### Examination of the capability of AuNCs@Mann toward target bacteria

The AuNCs@Mann (1.36 mg/mL, 0.1 mL) were vortex-mixed with a bacterial sample solution for 30 min and then quickly centrifuged at 2000 rpm for 15 s. The sample vial was placed under UV light illumination (λmax = 365 nm) for examination. To achieve optimized results, the bacterial samples were centrifuged at 3500 rpm for 5 min after vortex-mixed for 30 min–3 h.

### Analysis of urine samples spiked with *E. coli* J96

The urine samples used this study were obtained from a healthy individual. The consent of the subject was verbal. No personal or background information of this individual is disclosed. According to the Article 8 of the Medical Care Act (Taiwan), such urine samples are not regulated by the Regulations of Human Trials (Taiwan). This effectively deems ethics approval of this study unnecessary. The urine sample was used for making background complex and allowed us to investigate the feasibility of using our nanoprobes to selectively enrich target bacteria from such complex matrix. The urine sample centrifuged by a Hermle centrifuge (model: Z232 K, Munich, Germany) equipped with a rotor with the radius of 7.5 cm at 7000 rpm for 30 min. The supernatant was diluted 100–fold by PBS solution (pH 6.5). Urine samples spiked with *E. coli* J96 were prepared by spiking *E. coli* J96 to the diluted urine solution. The as-prepared urine sample containing *E. coli* J96 solution (OD600 = 1) was serial diluted 10–5000 fold by the diluted urine to prepare urine samples with difference cell concentrations of *E. coli* J96. The AuNCs@Mann (1.36 mg/mL, 0.1 mL) was added individually to the urine samples (0.9 mL) containing *E. coli* J96 with difference cell concentrations.

## Results and Discussion

**Figure 1:**
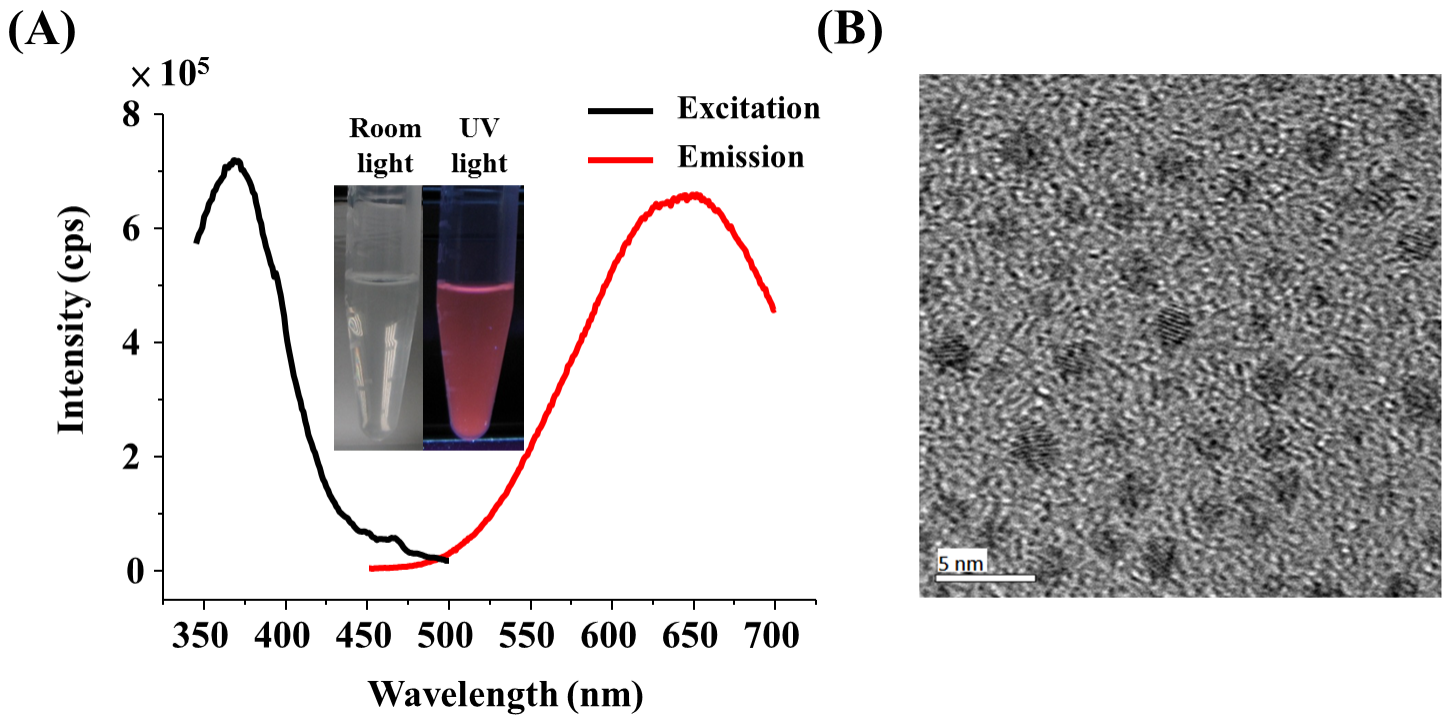
Characterization of the generated AuNCs@Mann. (A) Excitation and emission spectra (λ_excitation_ = 375 nm) of the generated AuNCs@Mann obtained from a Horiba Jobin Yvon FluoroMax-3 spectrofluorometer. The inset in Panel A is the photographs of the generated AuNCs@Mann obtained under room light (left) and ultraviolet light (right). (B) Transmission electron microscopic image of the as-prepared AuNCs@Mann. The scale bar is 5 nm.

Initially, we prepared the AuNCs@Mann via a one-step synthesis approach. Figure 1A presents the excitation and emission spectra of the generated AuNCs@Mann. The AuNCs@Mann have reddish photoluminescence with maximum emission at a wavelength of ∼630 nm (λ_excitation_ = 375 nm). The inset is the photograph of the AuNCs@Mann taken under room lighting and UV illumination. Figure S1 shows the absorption spectrum of AuNCs@Mann, in which two apparent absorption bands appear at the wavelengths of ∼220 nm and ∼340 nm. The quantum yield of the AuNCs@Mann was estimated to be ∼0.41% (Fig. S2). Figure 1B displays the image of the transmission electron microscopy (TEM) of the AuNCs@Mann. The size of the AuNCs@Mann is estimated at 1.95±0.27 nm. X-ray photoelectron spectroscopy (XPS) was also used to characterize the AuNCs@Mann. Figures S3A and S3B display the XPS spectra of Au 4f and S 2p of the AuNCs@Mann, respectively. A peak appearing at 84.8 eV (Au 4f7/2) results from the combination of the binding energy of Au (0) and Au (I), corresponding to 84.0 eV and 84.9 to 85.3 eV [16], respectively. The binding energy of S 2p at 163.0 eV was also observed in the XPS spectrum of the AuNCs@Mann (Fig. S3B), suggesting that the Mann-SH molecules were incorporated in the gold nanoclusters (Fig. S3B).

**Figure 2:**
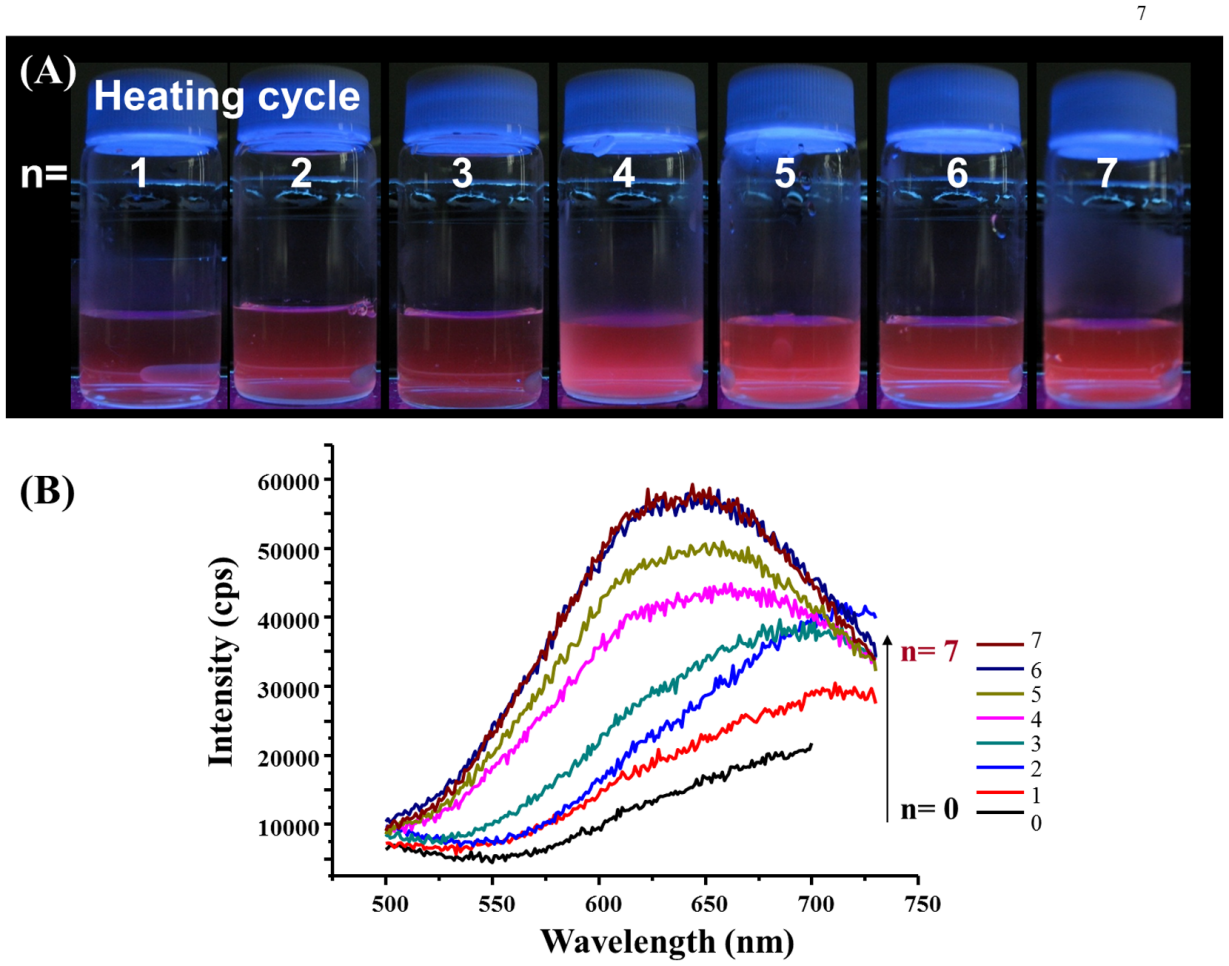
Generation of the AuNCs@Mann under microwave-heating. (A) and (B) display the photographs and corresponding emission spectra of the reaction products obtained after successive heating cycles [n, n = 1 to 7 (left to right), 5 min/heating cycle] in a domestic microwave oven (power: 90 W).

To expedite the preparation of the AuNCs@Mann, microwave heating was applied because such a method has been widely used for accelerating chemical reactions [17], [18] and synthesizing nanomaterials [19], [20]. Thus, 6-mercaptohexyl α-d-mannopyranoside (Mann-SH) (3 mM, 2 mL) was first stirred with tetrachloroauric acid (1 mM, 2 mL) for 10 min and resulting mixture was heated in a microwave oven with a low power (90 W). Figures 2A and 2B display the photographs and emission spectra corresponding to the mixture obtained in subsequent heating cycles [n, n = 1 to 7 (left to right), 5 min/heating cycle] (power: 90 W). The more the heating cycle, the more intense reddish photoluminence is observed. The intensity of the maximum emission band at the wavelength of ∼630 nm (λ_excitation_ = 375 nm) increased as the heating cycles increased (Fig. 2B). After seven heating cycles, the intensity of the maximum emission band did not change further, indicating the end of the reaction. The results illustrate that the AuNCs@Mann can be generated rapidly under microwave heating, significantly shortening the reaction time from 48 h to less than 1 h.

**Figure 3:**
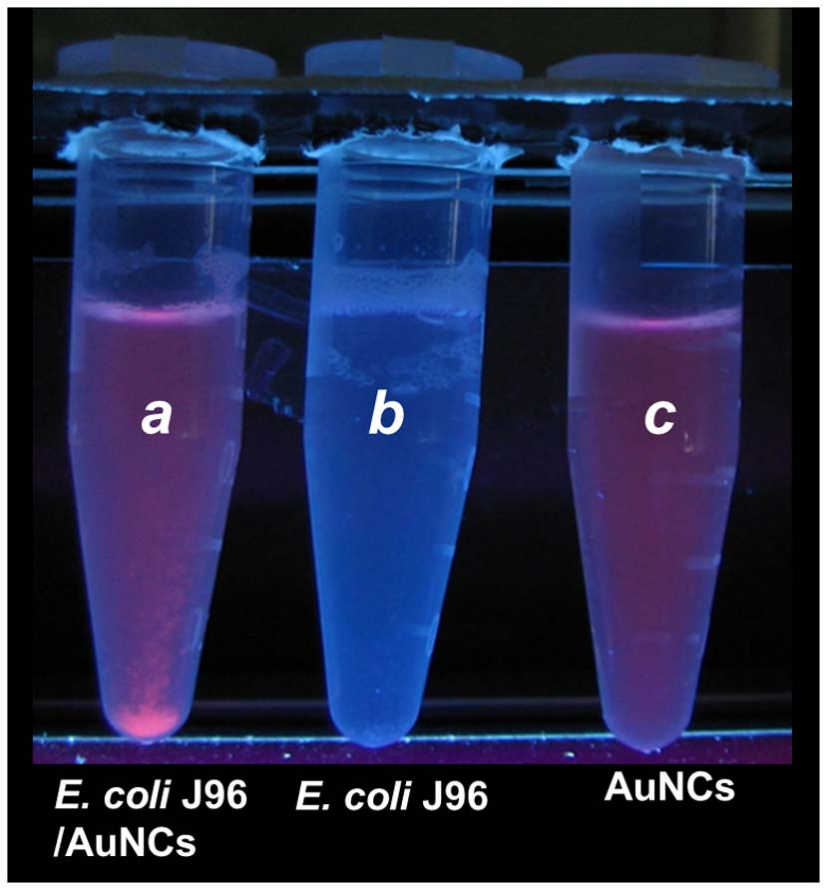
Using the AuNCs@Mann as the sensing probes for *E. coli* J96. Photograph of the samples obtained after using the AuNCs@Mann (1.36 mg/mL) as the sensor for the sample containing *E. coli* J96. Vials *a*, *b*, and *c* contained AuNCs@Mann/*E. coli* J96 (1.36 mg/mL, 0.1 mL/1.43×10^6^ cells/mL, 0.9 mL), *E. coli* J96 (1.29×10^6^ cells/mL, 1 mL), and AuNCs@Mann (0.136 mg/mL, 1 mL), respectively. The photograph was taken under UV light (λ_max_ = 365 nm).

We further examined the capability of the AuNCs@Mann for probing bacteria that contains mannose binding receptor proteins. *E. coli* J96, which contains the receptors, was chosen as the detection target. Initially, a yeast agglutination test was conducted to confirm the presence of the mannose binding receptors in freshly *E. coli* J96 before employing the bacterial sample for further experiments. The AuNCs@Mann (1.36 mg/mL, 0.1 mL) were vortex-mixed with a sample solution containing *E. coli* J96 [1.43×10^9^ cells/mL (OD_600_ = 1), 0.9 mL] for 30 min and then quickly centrifuged at 2,000 rpm for 15 s. Vial *a* in Figure 3 presents the resultant photograph taken under UV light illumination (λ_max_ = 365 nm). Precipitation with reddish photoluminescence was observed at the bottom of the vial under the UV illumination. However, the control samples containing *E. coli* J96 (vial *b*) and the AuNCs@Mann (vial *c*) conducted in parallel showed no sight of precipitation. The precipitation with reddish photoluminescence in vial *a* could be interpreted as a consequence of the binding of the AuNCs@Mann to *E. coli* J96. The binding of AuNCs@Mann to *E. coli* J96 increases the apparent weight of the bacteria, which led to the easy sedimentation of conjugates at the bottom of the vial *a* at a low centrifugation speed in a short period of time. The results indicate that the generated AuNCs@Mann can target *E. coli* J96 with mannose binding receptors. Additionally, we also examined *Staphylococcus aureus* to investigate the selectivity of the AuNCs@Mann. *S. aureus* contains no mannose binding sites and in theory would not bind the AuNCs@Mann. Figures S4A and S4B display the yeast agglutination test obtained by examining *E. coli* J96 and *S. aureus*, respectively. Agglutination occurred in the *E. coli* J96 sample, but not in the *S. aureus* sample, confirming the absence of the mannose binding receptors in the latter bacteria. Figure S4C shows the photograph obtained after vortex-mixing the *E. coli* J96 (left) and *S. aureus* (right) samples with the AuNCs@Mann followed by centrifugation. Precipitation with red photoluminescence was observed in the *E. coli* J96 sample mixed with the AuNCs@Mann (vial at the left hand side of Fig. S4C). No precipitation was observed the *S. aureus* sample (vial at the right hand side of Fig. S4C). Taken together the results indicate that the AuNCs@Mann binds selectively to bacterial targets containing mannose binding sites.

**Figure 4:**
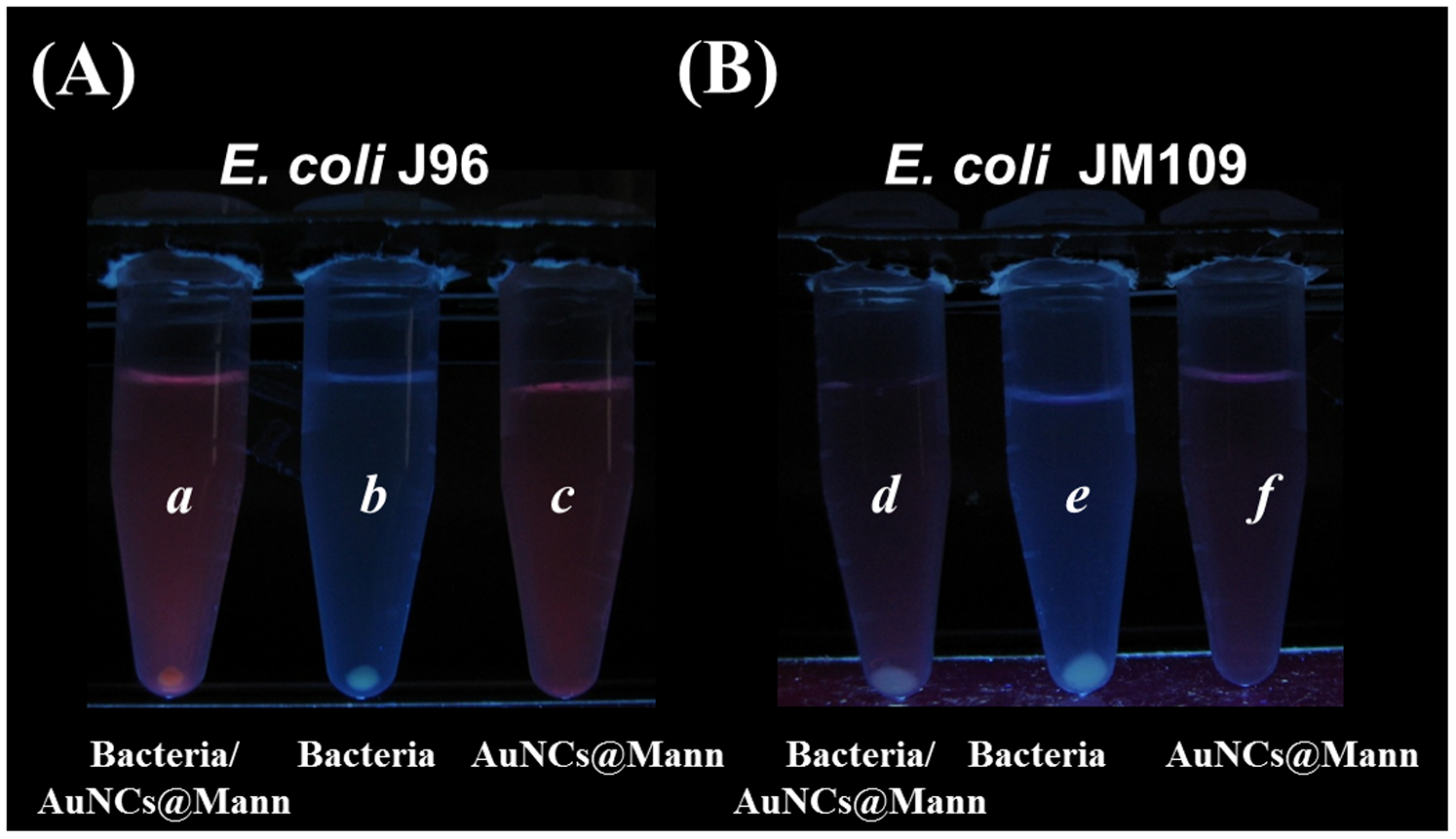
Using the AuNCs@Mann as the selective probes for different *E. coli* strains. Photographs obtained after using the AuNCs@Mann generated from microwave heating as the sensing probes for target bacteria under UV light (λ_max_ = 365 nm). (A) Vial *a* was obtained after vortex-mixing AuNCs (1.36 mg/ml, 0.1 mL) with a sample solution containing *E. coli* J96 (OD_600_ = 1, 0.9 mL) for 30 min, followed by centrifugation at 3,500 rpm for 5 min. Vials *b* and *c* were the control samples, which contained *E. coli* J96 (OD_600_ = 0.9, 1 mL) and AuNCs (0.136 mg/ml, 1 mL), respectively. (B) Vial *d* was obtained after vortex-mixing AuNCs (1.36 mg/ml, 0.1 mL) with a sample solution containing *E. coli* JM109 (OD_600_ = 1, 0.9 mL) for 30 min, followed by centrifugation at 3,500 rpm for 5 min. Vials *e* and *f* were the control samples, which contained *E. coli* JM109 ((OD_600_ = 0.9, 1 mL) and AuNCs (0.136 mg/ml, 1 mL), respectively.

Encouraged by the sensing studies above, we next examined if the AuNCs@Mann can differentiate *E. coli* strains with and without mannose binding receptors. To this regard, *E. coli* J96 containing mannose binding sites and *E. coli* JM109 lacking mannose binding receptors were selected as the models [21]. Figures S5A and S5B show the photographs of the samples obtained from the yeast agglutination test of *E. coli* J96 and *E. coli* JM109, respectively. Apparently, agglutination appeared in the *E. coli* J96 sample (Fig. S5A) but not in the *E. coli* JM109 sample (Fig. S5B), confirming the absence of mannose binding sites in *E. coli* JM109. AuNCs@Mann (1.36 mg/mL, 0.1 mL) was added to these two bacterial samples (0.9 mL, OD_600_ = 1) to repeat the bacterial sensing experiments. Two samples, one containing AuNCs@Mann only and the other with the bacterium sample only as control samples, were prepared in parallel. All these samples were vortex-mixed for 30 min and then centrifuged at 3,500 rpm for 5 min. Figures 4A and 4B display the resultant photographs of the sample vials containing *E. coli* J96 and *E. coli* JM109, respectively, which were taken under UV light (λ_max_ = 365 nm). Only the mixture of the *E. coli* J96 sample and AuNCs@Mann gave precipitation with reddish photoluminescence on the bottom of vial *a* (Fig. 4A). In the control sample with *E. coli* J96 alone (vial *b*, Fig. 4A), precipitation with light blue photoluminescence resulting from the auto-fluorescence of the bacterial cells was observed. However, no precipitation was observed in the vial *c*, which contained only the AuNCs@Mann implicating the stability of the AuNCs@Mann in the sensing experiment (vial *c*, Fig. 4A). For the sample containing both *E. coli* JM109 and AuNCs@Mann, precipitation with blue photoluminescence from the auto-fluorescence of the bacteria alone was observed on the bottom of the sample vial *d* (Fig. 4B). This result is attributed to the absence of mannose binding receptors in bacteria. Precipitation with blue photoluminescence was also observed at the bottom of the vial *e*, which contained only *E. coli* JM109. Moreover, no precipitation was observed on the control sample that contained only the AuNCs@Mann (vial *f*). The reddish AuNCs still suspend quite well in the solution. The results clearly indicate that the AuNCs@Mann in the current study can selectively bind with bacteria that contain mannose binding receptors.

To confirm the results further, we employed the optical microscopy to examine the precipitation obtained from the vials of Figure 4A. The precipitate was resuspended in PBS solution before examined. Figure S6A displays the optical microscopy image of the sample obtained from the precipitation of vial *a* shown in Figure 4A under bright field, whereas Figure S6B displays the image of the same sample under a fluorescence microscope (λ_excitation_ = 330 nm to 380 nm). Bacterial aggregations were observed in both images. The red photoluminescence shown in Figure S6B indicates that AuNCs were attached to *E. coli* J96 leading to aggregations. Figure S6C shows the optical microscopy image of the precipitation obtained from vial *b* shown in Figure 4A under bright field. Figure S6D shows the image of the same sample as in Figure S6C under a fluorescence microscope (λ_excitation_ = 330 nm to 380 nm). Unlike the precipitate examined in Figures S6A and S6B, those in Figure S6C does not aggregate on the glass slide. The bacteria have no apparent emission under the UV illumintation (Fig. S6D). The results confirm that the AuNCs@Mann can interact with *E. coli* J96, resulting in aggregations.

**Figure 5:**
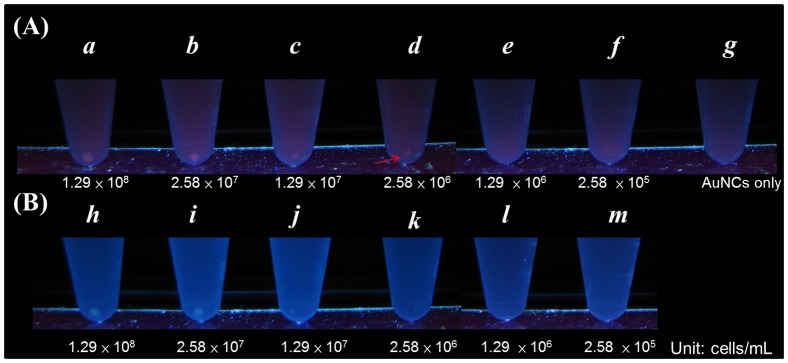
Observation by the naked eye. Photographs obtained after using the AuNCs@Mann generated from microwave heating as the sensing probes for urine sample spiked with different cell concentrations of *E. coli* J96 under UV light (λ_max_ = 365 nm). (A) Vials *a–g* were obtained after vortex-mixing the AuNCs@Mann (1.36 mg/ml, 0.1 mL) with 100-fold diluted urine (0.9 mL) containing *E. coli* J96 with different cell concentrations for 30 min, followed by centrifugation at 3,500 rpm for 5 min. The cell concentrations of *E. coli* J96 in vials *a–g* were 1.29×10^8^ cells/mL, 2.58×10^7^ cells/mL, 1.29×10^7^ cells/mL, 2.58×10^6^ cells/mL, 1.29×10^6^ cells/mL, 2.58×10^5^ cells/mL, and 0 cells/mL, respectively. (B) Vials *h–m* were the control samples, which only contained *E. coli* J96 with the cell concentrations of 1.29×10^8^ cells/mL, 2.58×10^7^ cells/mL, 1.29×10^7^ cells/mL, 2.58×10^6^ cells/mL, 1.29×10^6^ cells/mL, 2.58×10^5^ cells/mL, respectively.

The limit of detection of *E. coli* J96 using this approach was evaluated. Figure 5 presents the photograph obtained from the sensing results using the AuNCs@Mann as the sensors for urine samples spiked with *E. coli* J96 at different concentrations. After the urine samples (*a–m*) were vortex-mixed with the sensing probes for 30 min, followed by centrifugation at 3,500 rpm for 5 min, the reddish photoluminescence resulting from the *E. coli* J96-AuNCs@Mann conjugates was clearly observed at the bottom of the vials *a–d*, which contained the *E. coli* J96 cells≧ 2.58×10^6^ cells/mL. No precipitation was observed in vials *e* and *f*, which contained 1.29×10^6^ and 2.58×10^5^ *E. coli* J96 cells/mL, respectively. Furthermore, no precipitation was observed on the bottom of vial *g*, which contained only AuNCs@Mann. The results indicate that the current method may be used to visualize the presence of *E. coli* J96 in the sample containing ∼2.58×10^6^ of cells/mL (vial *d*). In the absence of the AuNCs@Mann (vials *h–m* in Fig. 5), it is barely seen the presence of the bacterial cells on the bottom of the sample vials after a low speed of centrifugation even the bacterial cell concentration was as high as ∼1.29×10^7^ cells/mL (vial *j*). The results indicate that the present approach of bacteria detection can be used to visualize the presence of *E. coli* J96 with desirable sensitivity. Although the limit of detection of this approach is not particularly low, this method provides a fast track for screening of *E. coli* J96 in urine samples. To demonstrate the feasibility of using our method to quantitative analysis, fluorescence spectroscopic analysis was performed. Figure S7A shows the fluorescence spectra of the supernatant obtained after vortex-mixing of the AuNCs@Mann and *E. coli* J96 for 30 min followed by centrifugation at 3500 rpm for 30 min. The centrifugation time was longer than shown in other experiments to ensure all the bacterial cells were settled down on the bottom of the tubes. The fluorescence intensity of the supernatant decreased as the increase of the cell concentration of *E. coli* J96 in the sample solution because more AuNCs@Mann was bound by *E. coli* J96 and subsequently spun down as precipitate upon centrifugation. The fluorescence spectrum obtained from the AuNCs@Mann (0 cells/mL) was almost overlapped with the lowest cell concentration at 1.4 (10^5^ cells/mL. However, we can still distinguish the difference of the fluorescence spectrum of the blank from the *E. coli* J96 sample with the cell concentration at ∼2.8 (10^5^ cells/mL, which is about one order of magnitude lower than that observed by the naked-eye detection (Fig. 5). Figure S7B shows the plot of the fluorescence intensity difference at 670 nm between the bacterial sample and blank solution obtained in Figure S7A versus different cell concentrations. The results indicated that it is possible to conduct quantitative analysis using fluorescence spectroscopy as the detection tool.

Additionally, we also compared the detection sensitivity of the conventional yeast agglutination test [22] with our method. Figure S8 displays the results of the agglutination test obtained by examining the samples of *E. coli* J96 at different cell concentrations. When the cell concentration was at 1.29×10^9^ cells/mL, apparent agglutination was observed (row 1, Fig. S8) in the *E. coli* J96/yeast droplet. However, when the cell concentration of *E. coli* J96 decreased to 1.29×10^8^ cells/mL, and no obvious agglutination was observed in the *E. coli* J96/yeast droplet (row 2, Fig. S8). Therefore, the approximate detection limit of the agglutination test for *E. coli* J96 is at around 1.29×10^9^ cells/mL. The results indicate that the yeast agglutination test is not as sensitive as our method (*cf.* Fig. 5). Thus, the AuNCs@Mann can be used to screen the presence of target species containing mannose binding sites with a higher sensitivity.

## Conclusions

We have demonstrated a facile way to generate mannose-capped AuNCs with reddish photoluminescence. The functional AuNCs can be generated within 1 h with the assistance of microwave-heating. The reaction time was considerably shorter than those reported previously, and the generated AuNCs@Mann still remain their capability of interacting with target bacteria containing mannose-binding receptors. The current approach simply used the naked eye as the detection method and did not require any sophisticated instruments. Additionally, the selectivity of the functional AuNCs for target bacteria containing mannose binding sites is quite good. Bacteria with/without mannose binding sites can be easily distinguished using this current sensing approach with desirable sensitivity. On the basis of the synthesis method and sensing approach proposed in this work, it is possible to generate different glycan-capped AuNCs using the microwave-heating assisted synthesis method within a short period of time. Microbial biosensors for various types of bacteria containing different glycan binding sites therefore can be developed based on the approach proposed in this work.

## Supporting Information

Figure S1 **Optical property of AuNCs@Mann.** Absorption spectrum of AuNCs@Mann.
(TIF)

Figure S2 **Estimation of the quantum yield (QY) of the AuNCs@Mann.** Rbioflavin-5′ phosphate (RFMP) was used as the standard (QY: 26%). The excitation wavelength was set at 370 nm. The integrated area (***F***) of the resultant emission band obtained from different concentrations was plotted in Y axis, while the absorption value (***A***) at the wavelength of 370 nm obtained from different concentrations was plotted in X axis. Panel A shows the correlation curve by plotting ***F*** versus ***A*** using RFMP as the reference standard, and Panel B shows the correlation curve by plotting F versus A using AuNCs@Mann as the sample. (C) The QY of the AuNCs@Mann (B) was estimated ∼0.41%.
(TIF)

Figure S3 **X-ray photoelectron spectroscopy (XPS) spectral analysis.** XPS spectra of (A) Au 4f and (b) S 2p for AuNCs@Mann (C 1s binding energy (285.3 eV) was used as an internal reference).
(TIF)

Figure S4 **Selectivity test of AuNCs@Mann for target bacteria.** Photographs obtained from the yeast agglutinin test of (A) *E. coli* J96 and (B) *S. aureus*. (C) Photograph obtained after vortex-mixing the AuNCs@Mann (0.136 mg/mL) with the bacterial samples for 30 min followed by centrifugation at 2000 rpm for 30 s. *E. coli* J96 (OD = 0.9) (left) and *S. aureus* (OD = 0.9) (right) were prepared in diluted urine/PBS buffer (pH 6.5) (1∶100, v/v).
(TIF)

Figure S5 **Yeast agglutination test for different** ***E. coli*** **strains.** Photographs obtained from the yeast agglutinin test of (A) *E. coli* J96 and (B) *E. coli* JM109.
(TIF)

Figure S6 **Microscope images.** (A) Optical microscope image of the sample obtained from the precipitation in vial a in Figure 4A under bright field and (B) the image of the same sample obtained under a fluorescence microscope (λ_excitation_ = 330–380 nm) (Nikon, model: Eclipse 80i). (C) Optical microscope image of the sample obtained from the precipitation in vial *b* in Figure 4A under bright field and (D) the image of the same sample obtained under a fluorescence microscope (λ_excitation_ = 330 nm to 380 nm) (Nikon, model: Eclipse 80i).
(TIF)

Figure S7 **Fluorescence spectroscopic analysis.** (A) Fluorescence spectra of the supernatants obtained after vortex-mixing the AuNCs@Mann(1.35 mg/mL, 0.1 mL) with *E. coli* J96 (0.9 mL) at the cell concentrations of 2.8×10^8^ (red), 1.4×10^8^ (navy-blue), 2.8×10^7^ (pink), 1.4×10^7^ (grass green), 2.8×10^6^ (green), 1.4×10^6^ (indigo), 2.8×10^5^ (brown), 1.4×10^5^ (pink-red), and 0 (blank, black) cells/mL in PBS buffer (pH 6.5). (B) Plot obtained from Panel A by plotting the intensity difference between the supernatant and the blank sample (0 cells/mL) at the wavelength of 670 nm (λ_max_ = 375 nm). I_s_ stands for the intensity (λ_emi_ = 670 nm) of the sample, while I_0_ stands for the intensity (λ_emi_ = 670 nm) of the blank sample only containing the AuNCs@Mann.
(TIF)

Figure S8 **Yeast agglutination test of** ***E. coli*** **J96 at different concentrations.** Yeast agglutinin test results obtained by using different cell concentrations of *E. coli* J96 as the samples.
(TIF)

File S1 **Procedure for the preparation of Mann-SH.**
(DOC)
