# Supplementary figures and images for: Photoluminescent Gold Nanoclusters as Sensing Probes for Uropathogenic Escherichia coli
Source: PLoS One. 2013 Mar 15;8(3):e58064. doi: 10.1371/journal.pone.0058064 (PMC3598911; doi:10.1371/journal.pone.0058064)

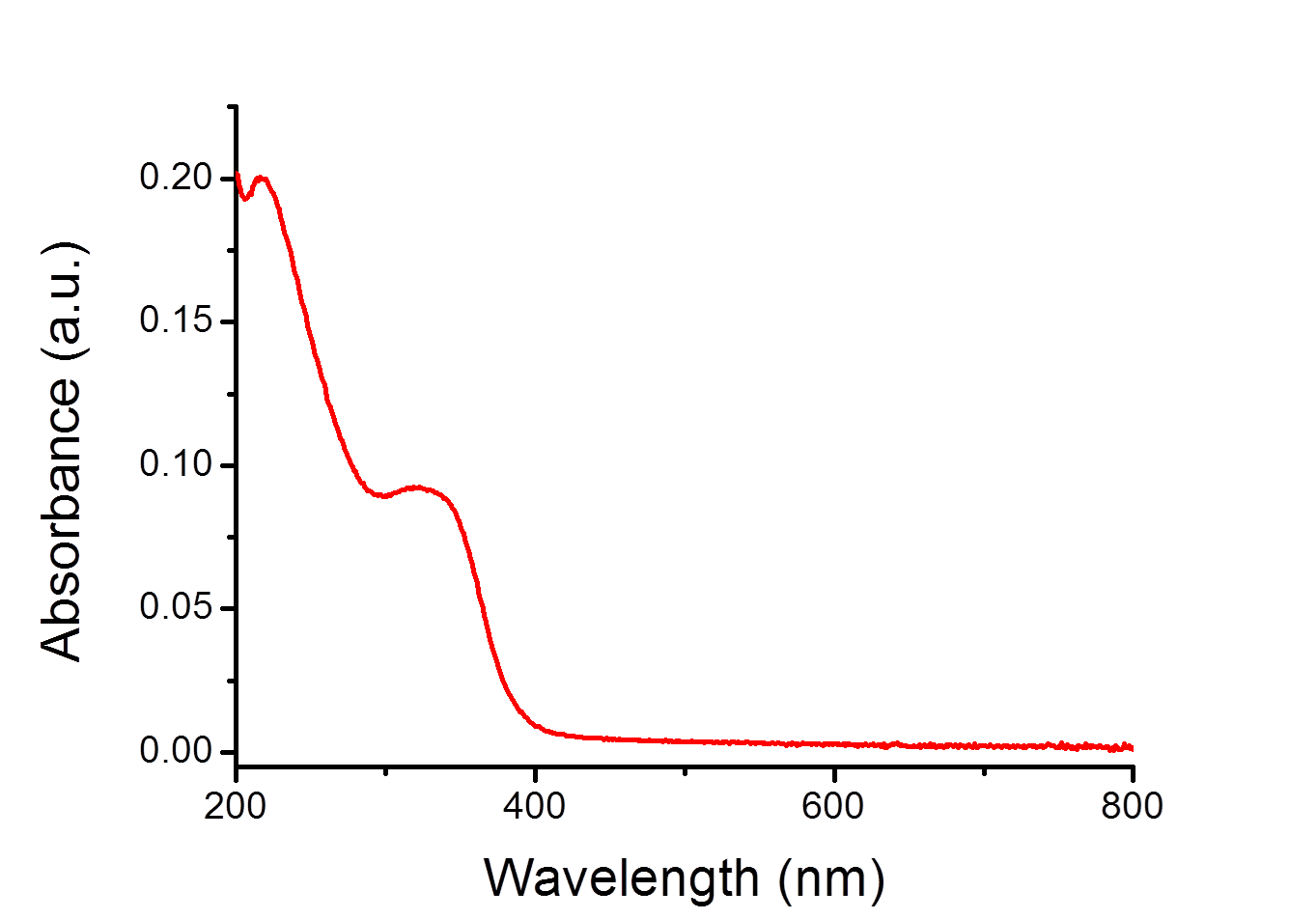

Supplement: Figure S1 — Optical property of AuNCs@Mann. Absorption spectrum of AuNCs@Mann. (TIF) [file pone.0058064.s001.tif]

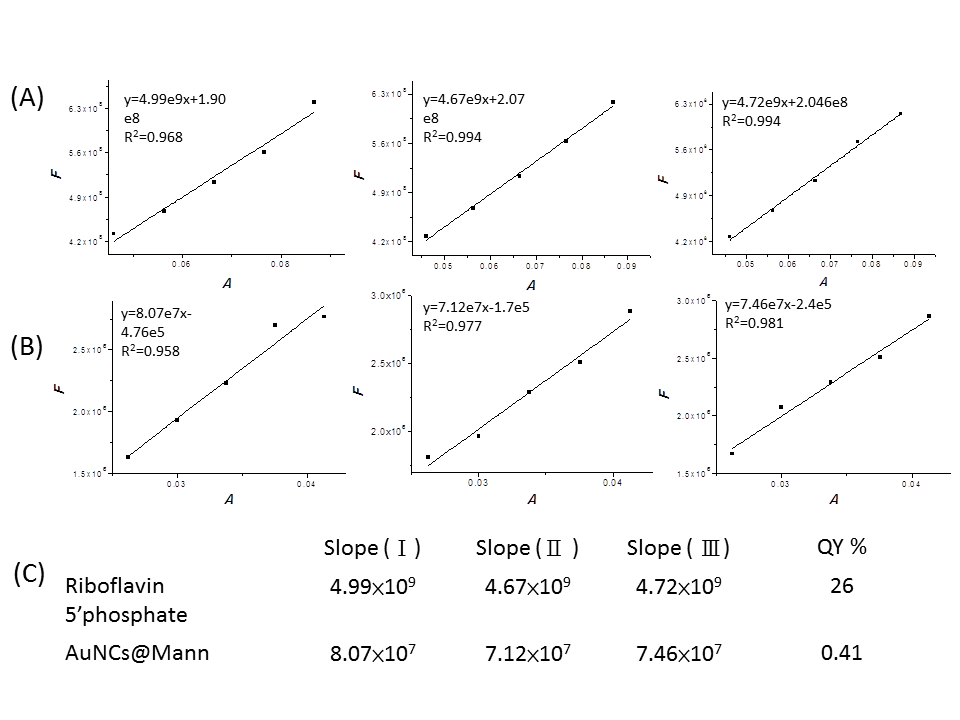

Supplement: Figure S2 — Estimation of the quantum yield (QY) of the AuNCs@Mann. Rbioflavin-5′ phosphate (RFMP) was used as the standard (QY: 26%). The excitation wavelength was set at 370 nm. The integrated area (F) of the resultant emission band obtained from different concentrations was plotted in Y axis, while the absorption value (A) at the wavelength of 370 nm obtained from different concentrations was plotted in X axis. Panel A shows the correlation curve by plotting F versus A using RFMP as the reference standard, and Panel B shows the correlation curve by plotting F versus A using AuNCs@Mann as the sample. (C) The QY of the AuNCs@Mann (B) was estimated ∼0.41%. (TIF) [file pone.0058064.s002.tif]

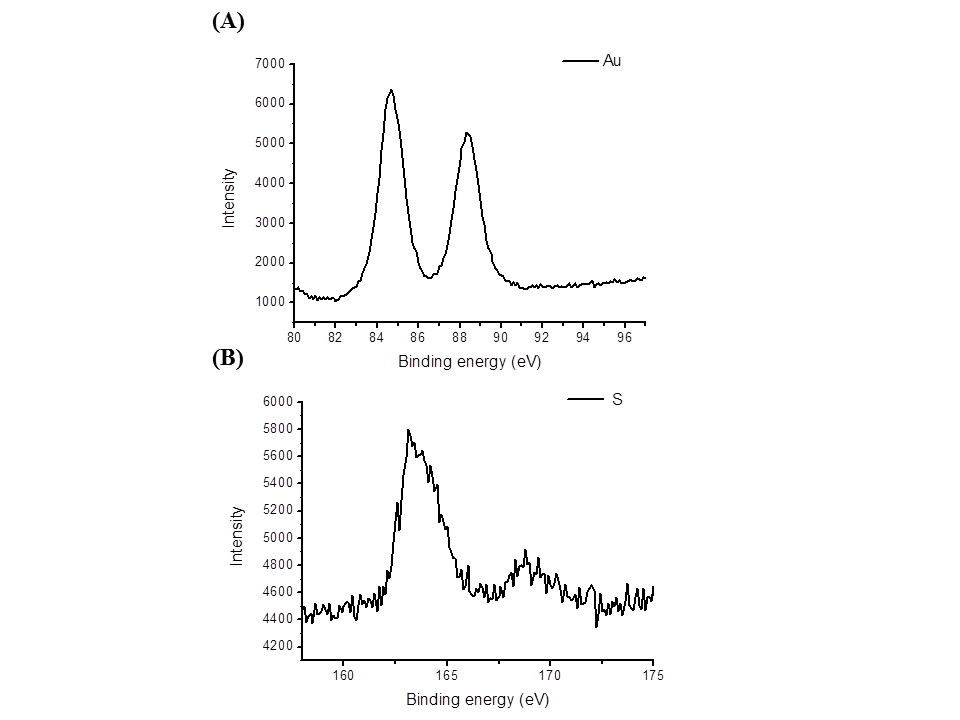

Supplement: Figure S3 — X-ray photoelectron spectroscopy (XPS) spectral analysis. XPS spectra of (A) Au 4f and (b) S 2p for AuNCs@Mann (C 1s binding energy (285.3 eV) was used as an internal reference). (TIF) [file pone.0058064.s003.tif]

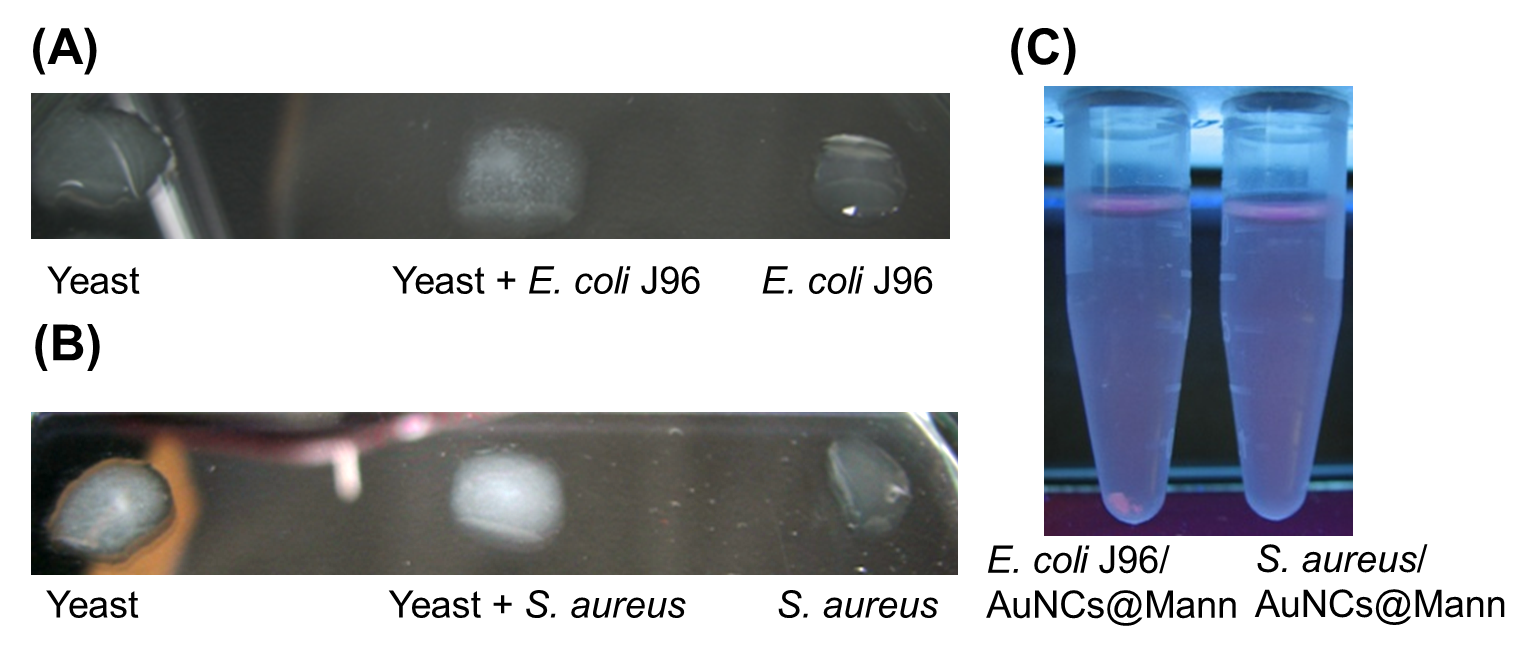

Supplement: Figure S4 — Selectivity test of AuNCs@Mann for target bacteria. Photographs obtained from the yeast agglutinin test of (A) E. coli J96 and (B) S. aureus. (C) Photograph obtained after vortex-mixing the AuNCs@Mann (0.136 mg/mL) with the bacterial samples for 30 min followed by centrifugation at 2000 rpm for 30 s. E. coli J96 (OD = 0.9) (left) and S. aureus (OD = 0.9) (right) were prepared in diluted urine/PBS buffer (pH 6.5) (1∶100, v/v). (TIF) [file pone.0058064.s004.tif]

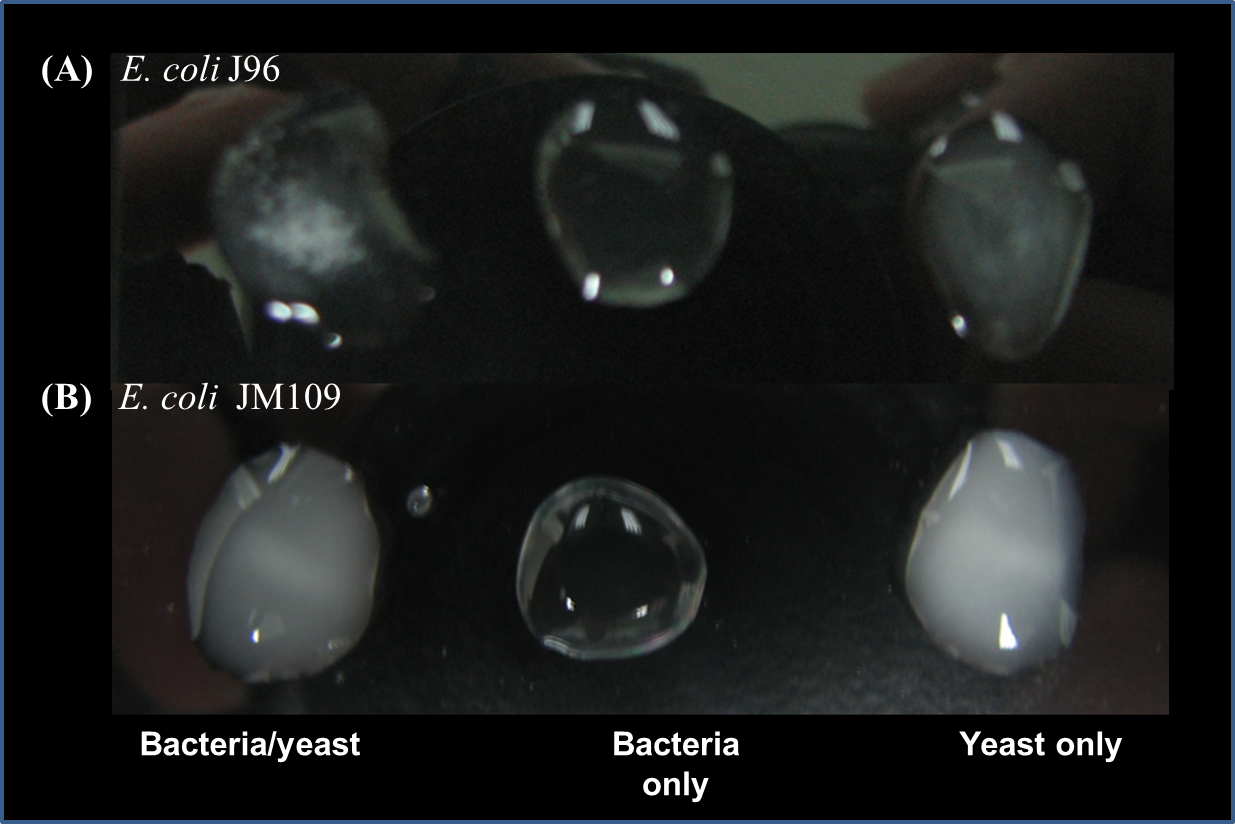

Supplement: Figure S5 — Yeast agglutination test for different E. coli strains. Photographs obtained from the yeast agglutinin test of (A) E. coli J96 and (B) E. coli JM109. (TIF) [file pone.0058064.s005.tif]

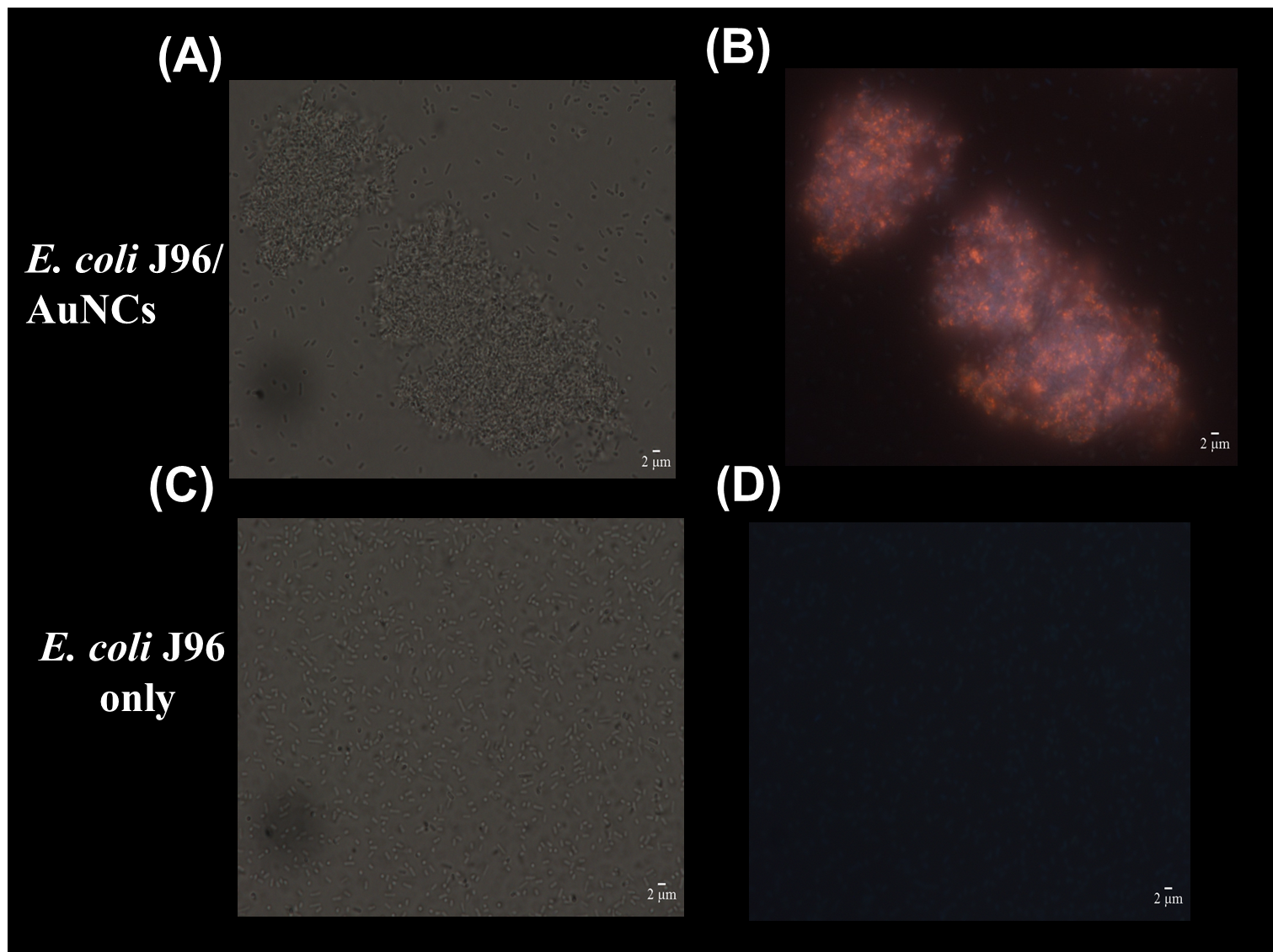

Supplement: Figure S6 — Microscope images. (A) Optical microscope image of the sample obtained from the precipitation in vial a in Figure 4A under bright field and (B) the image of the same sample obtained under a fluorescence microscope (λexcitation = 330–380 nm) (Nikon, model: Eclipse 80i). (C) Optical microscope image of the sample obtained from the precipitation in vial b in Figure 4A under bright field and (D) the image of the same sample obtained under a fluorescence microscope (λexcitation = 330 nm to 380 nm) (Nikon, model: Eclipse 80i). (TIF) [file pone.0058064.s006.tif]

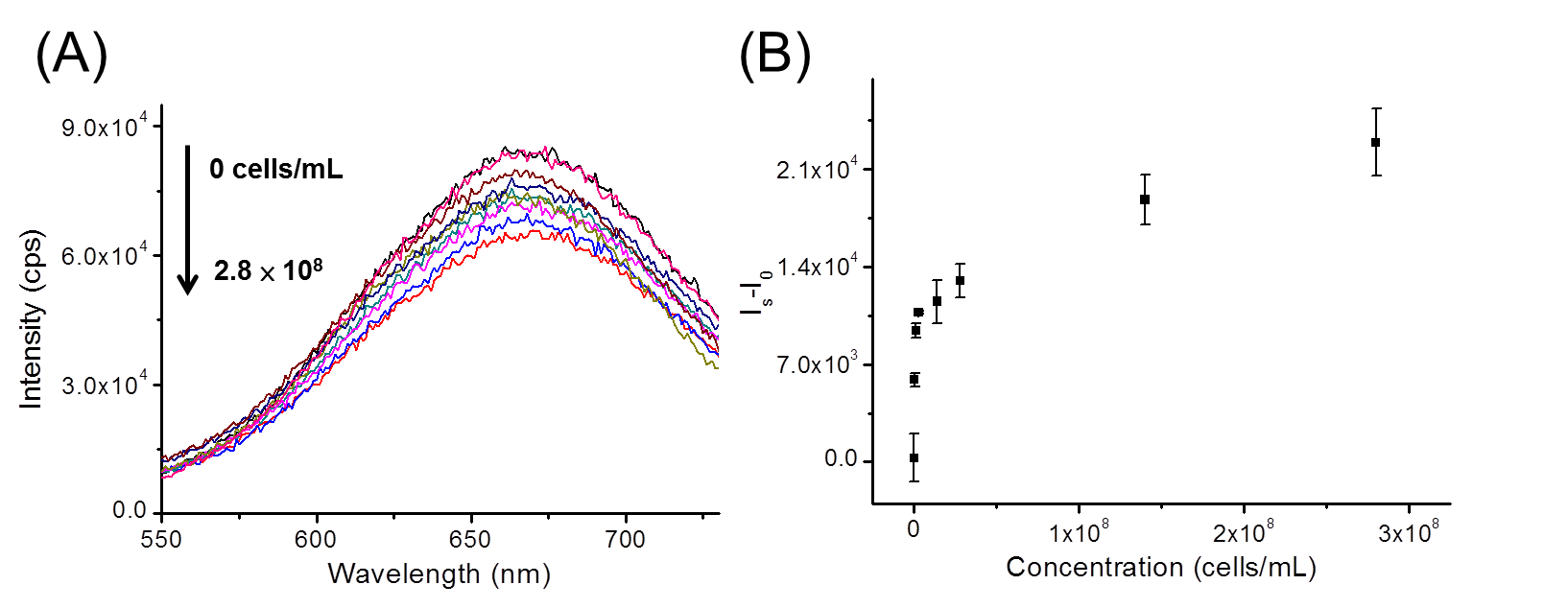

Supplement: Figure S7 — Fluorescence spectroscopic analysis. (A) Fluorescence spectra of the supernatants obtained after vortex-mixing the AuNCs@Mann(1.35 mg/mL, 0.1 mL) with E. coli J96 (0.9 mL) at the cell concentrations of 2.8×10^8^ (red), 1.4×10^8^ (navy-blue), 2.8×10^7^ (pink), 1.4×10^7^ (grass green), 2.8×10^6^ (green), 1.4×10^6^ (indigo), 2.8×10^5^ (brown), 1.4×10^5^ (pink-red), and 0 (blank, black) cells/mL in PBS buffer (pH 6.5). (B) Plot obtained from Panel A by plotting the intensity difference between the supernatant and the blank sample (0 cells/mL) at the wavelength of 670 nm (λmax = 375 nm). Is stands for the intensity (λemi = 670 nm) of the sample, while I0 stands for the intensity (λemi = 670 nm) of the blank sample only containing the AuNCs@Mann. (TIF) [file pone.0058064.s007.tif]

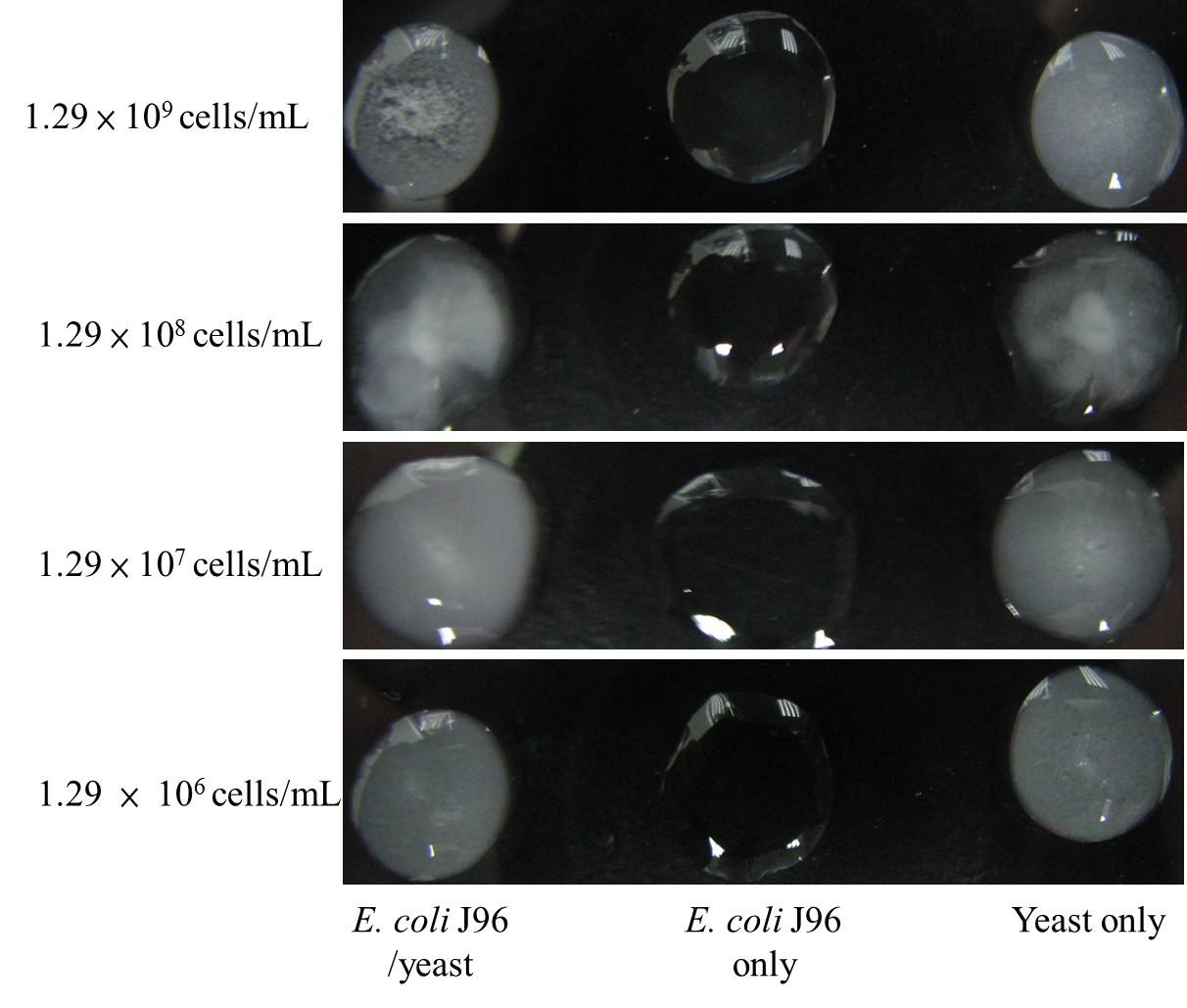

Supplement: Figure S8 — Yeast agglutination test of E. coli J96 at different concentrations. Yeast agglutinin test results obtained by using different cell concentrations of E. coli J96 as the samples. (TIF) [file pone.0058064.s008.tif]
