# Supplementary material for: Photoluminescent Gold Nanoclusters as Sensing Probes for Uropathogenic Escherichia coli
Source: PLoS One. 2013 Mar 15;8(3):e58064. doi: 10.1371/journal.pone.0058064 (PMC3598911; doi:10.1371/journal.pone.0058064)
Supplement: File S1 — Procedure for the preparation of Mann-SH. (DOC) [file pone.0058064.s009.doc]

**Photoluminescent Gold Nanoclusters as Sensing Probes for** **Uropathogenic *Escherichia coli***

Po-Han Chan,^a^ Bhaswati Ghosh,^a^ Hong-Zheng Lai, ^a^ Hwei-Ling Peng,^b^ Kwok Kong Tony Mong,^a*^ Yu-Chie Chen^a*^

^a^Department of Applied Chemistry, National Chiao Tung University, Hsinchu 300, Taiwan

^b^Department of Biological Science and Technology, National Chiao Tung University, Hsinchu 300, Taiwan

**Supporting Information**

**Synthesis of 6-Mercaptohexy-α-D-mannopyranoside**

**For 6-chlorohexyl 2,3,4,6-tetra-*O*-acetyl-α-D-mannopyranoside** **1a**: To a suspension of D-mannose (15.0 g, 83.3 mmol) in 3:1 Ac_2_O-CH_3_CN (80 mL) was added catalytic amount of TsOH (1.6 g, 8.3 mmol) at 0 ˚C. After stirring for 1 h, the temperature was gradually raised to RT and stirring was continued till completion of acetylation as assessed by TLC (~10 h). The reaction mixture was diluted with cold EtOAc (500 mL), washed with cold satd NaHCO_3_ (500 mL × 3), brine (300 mL × 1), dried (over MgSO_4_), filtered, and then concentrated to furnish per-*O*-acetyl mannopyranoside (32.5 g, quantitative). To a suspension of per-*O*-acetyl mannopyranoside (21.28 g, 54.6 mmol), 6-chlorohexanol (11.0 mL, 81.8 mmol) and flame-dried molecular sieves (AW300, 3.0 g) in CH_2_Cl_2_ (70 mL) was added BF_3_^.^Et_2_O (28 mL, 218 mmol). Followed by warming to 40 ˚C and stirred for 10 hours. The mixture was diluted with CH_2_Cl_2_ (500 mL), and filtered over celite. The resulting filtrate was washed with cold satd NaHCO_3_ (500 mL × 3), brine (300 mL × 1), dried (over MgSO_4_), filtered, and then concentrated for flash column chromatography (elution mixture: EtOAc/Hexane = 2/3) to furnish α-mannopyranoside **1a** (16.3 g, 58%) as a yellowish oil. For compound **1a**, *R*_f_ = 0.41 (EtOAc/Hexane = 2/3); ^1^H NMR (300 MHz, CDCl_3_): δ = 5.37−5.22 (m, 3 H), 4.80 (d, *J* = 1.5 Hz, 1 H, H-1), 4.01 (dd, *J* = 6.0, 5.4 Hz, 1 H, H-6), 4.00-3.95 (m, 1 H), 3.71-3.66 (m, 1 H, H-5), 3.55 (t, *J* = 6.6 Hz, 2 H, CH_2_Cl), 3.49-3.41 (m, 1 H, OCH_2_), 2.16 (s, 3 H, CH_3_CO), 2.08 (s, 3 H, CH_3_CO), 2.05 (s, 3 H, CH_3_CO), 2.00 (s, 3 H, CH_3_CO), 1.80-1.75 (m, 2 H, CH_2_), 1.68-1.59 (m, 2 H, CH_2_), 1.52-1.39 (m, 4 H, CH_2_); ^13^C NMR (75 MHz, CDCl_3_): δ = 171.1 (*C*=O), 170.5 (*C*=O), 170.3 (*C*=O), 170.1 (*C*=O), 97.9 (C-1), 70.1, 69.5, 68.8, 68.7, 66.6, 62.9, 45.4 (CH_2_Cl), 32.8, 29.5, 25.8, 21.3 (*C*H_3_CO), 21.1 (*C*H_3_CO), 21.1 (*C*H_3_CO),; HRMS−FAB (*m*/*z*): [M]^+^ calcd for C_20_H_31_ClO_10_, 466.1606; found, 466.1604.

**For 6-thioacetylhexyl 2,3,4,6-tetra-*O*-acetyl-α-D-mannopyranoside** **1b**: To a solution of **1a** (1 g, 33.08 mmol) in DMF (5 mL) was added potassium thioacetate (1.1 g, 99 mmol) and cesium carbonate (1.07 g, 33.08 mmol) at 0 ˚C and then stirred at room temperature. After 6 h, the substitution was complete as indicated by ^13^C NMR spectroscopy of crude reaction mixture, DMF was removed under reduced pressure. The residue was absorbed in EtOAc solution (300 mL), washed with 10% HCl (aq) (300 mL × 2 ), brine (300 mL × 1), dried (over MgSO_4_), filtered and then concentrated for a short pad silica gel chromatography (elution mixture: EtOAc/Hexane = 2/3) affording desired product **1b** (700 mg, 82%). For compound **1b**: *R*_f_ = 0.41 (EtOAc/Hexane = 1/1); ^1^H NMR (300 MHz, CD_3_OD): δ = 5.36−5.22 (m, 3 H), 4.80 (s, 1 H, H-1), 4.29 (dd, *J* = 9.57 Hz , 1 H), 4.10 (dd, *J* = 12.2 Hz, 1 H), 3.99 (m, 1 H ), 3.71-3.64 (m, 1 H )3.48-3.41 (m, 1 H), 2.87 (t, *J* = 10.95 Hz , 2 H), 2.33 (s, 3 H ), 2.16 (s, 3 H ), 2.11 (s, 3 H ), 2.05 (s, 3 H ), 1.99 (s, 3 H ), 1.61-1.57 (m, 4 H ), 1.39-1.37 ( m, 4 H ); ^13^C NMR (75 MHz, CDCl_3_): δ = 196.4 (*C*=O), 171.0 (*C*=O), 170.5 (*C*=O), 170.3 (*C*=O), 170.1 (*C*=O), 97.9 (C-1), 70.0, 69.5, 68.8, 68.7, 66.6, 62.9, 31.0, 29.8, 29.5, 29.4, 28.8, 26.0, 21.3 (*C*H_3_CO), 21.1(*C*H_3_CO), 21.1 (*C*H_3_CO); MS-FAB (m/z): [M + H]^+^ calcd for C_22_H_34_O_11_S, 506.18; found, 507.17.

**For 6-mercaptohexyl α-D-mannopyranoside** **1**: A solution of peracetyl mannopyranoside **1b** (700 mg, 23.0 mmol) in 1:2 CH_2_Cl_2_-MeOH mixture (7 mL) was treated with a piece of freshly cut sodium (ca. 50 mg) and stirred at RT and pH = ca 9 (pH paper). (Noted pH > 10 leaded to formation of disulfide). Upon completion of deacetylation, reaction mixture was neutralized with resin IR-120 H^+^, filtered, concentrated to furnish desired mannosyl ligand **1** (400 mg, > 94%) as off white solid. For compound **1**, *R*_f_ = 0.35 (CH_2_Cl_2_/MeOH = 10/1); ^1^H NMR (300 MHz, CD_3_OD): δ = 4.76 (s, 1 H, H-1), 3.79-3.40 (m, 9 H), 3.26 (m, 1 H) 2.44 (t, *J=*10.5 Hz, 2 H), 1.54 (m, 4 H ), 1.37-1.35 (m, 4 H ); ^13^C NMR (75 MHz, CD_3_OD): δ = 102.4 (C-1), 75.4, 73.5, 73.1, 69.4, 69.3, 63.8, 36.0, 31.3, 30.0, 27.7, 25.8; MS-FAB (m/z): [M + H]^+^ calcd for C_12_H_24_O_6_S, 296.13; found, 297.13.

^1^H NMR spectrum of 6-chlorohexyl 2,3,4,6-tetra-*O*-acetyl-α-D-mannopyranoside **1a**

*
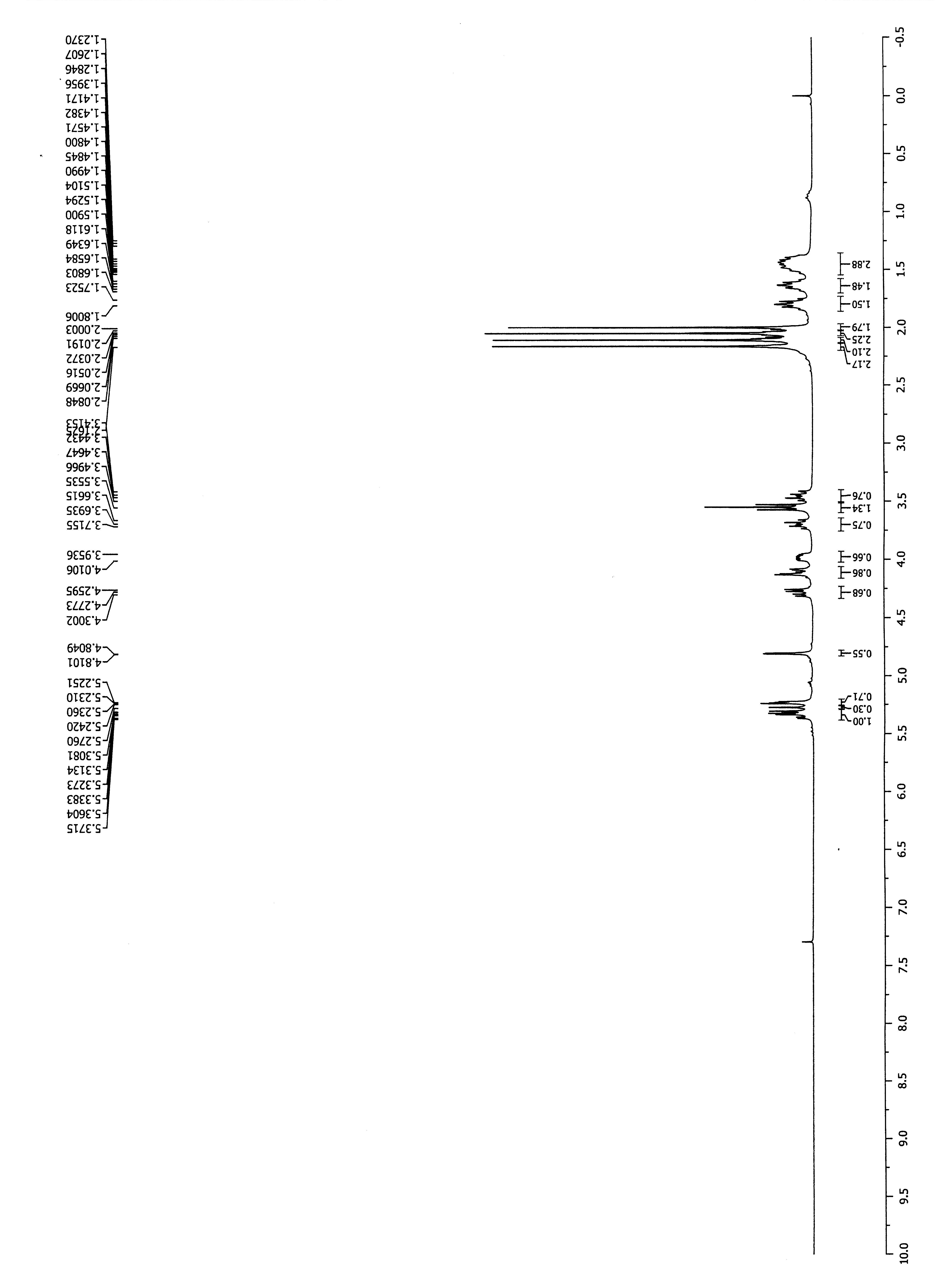
*

^13^C NMR spectrum of 6-chlorohexyl 2,3,4,6-tetra-*O*-acetyl-α-D-mannopyranoside **1a**

*
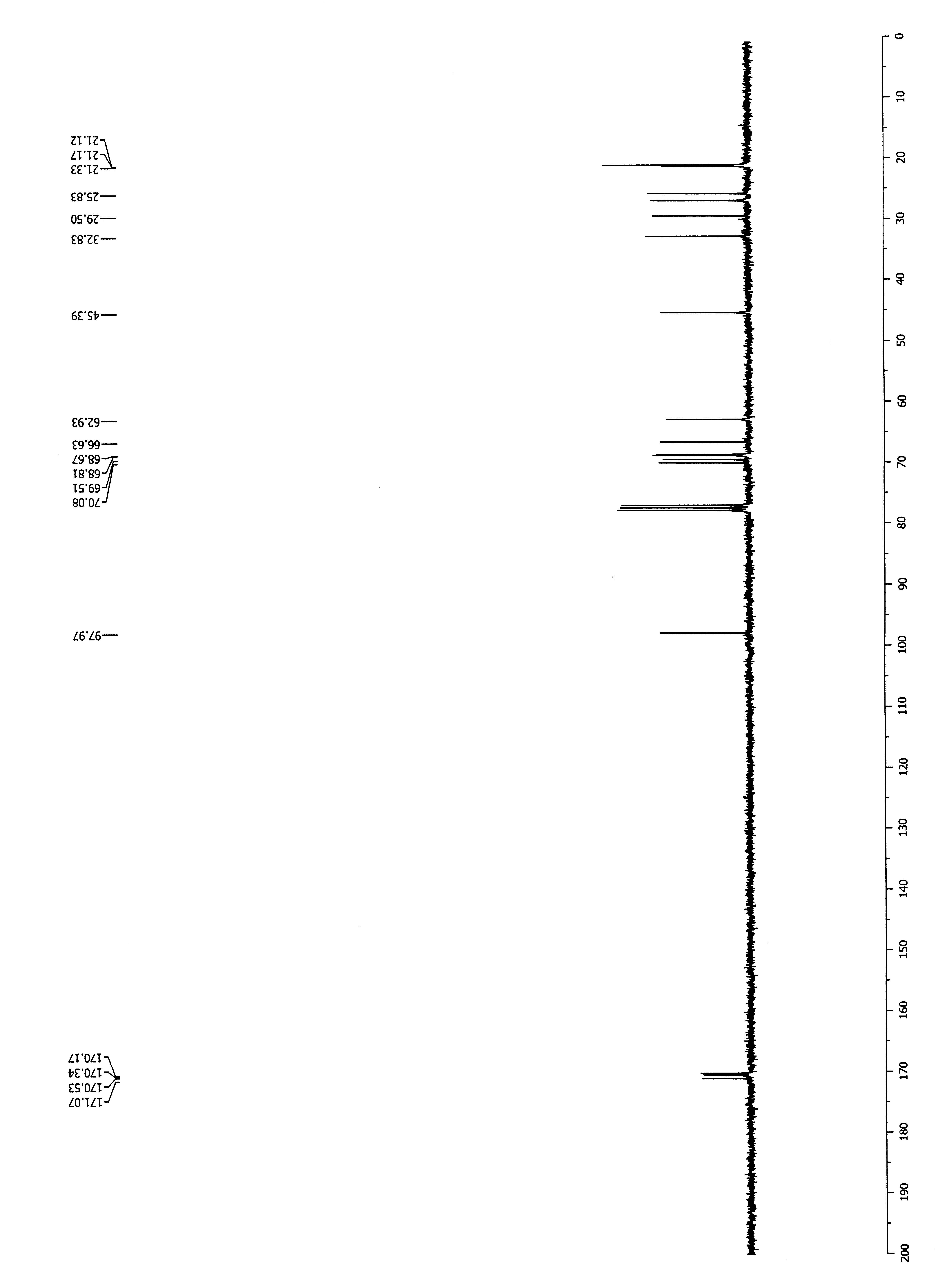
*

^1^H NMR spectrum of 6-thioacetylhexyl 2,3,4,6-tetra-*O*-acetyl-α-D-mannopyranoside **1b**

*
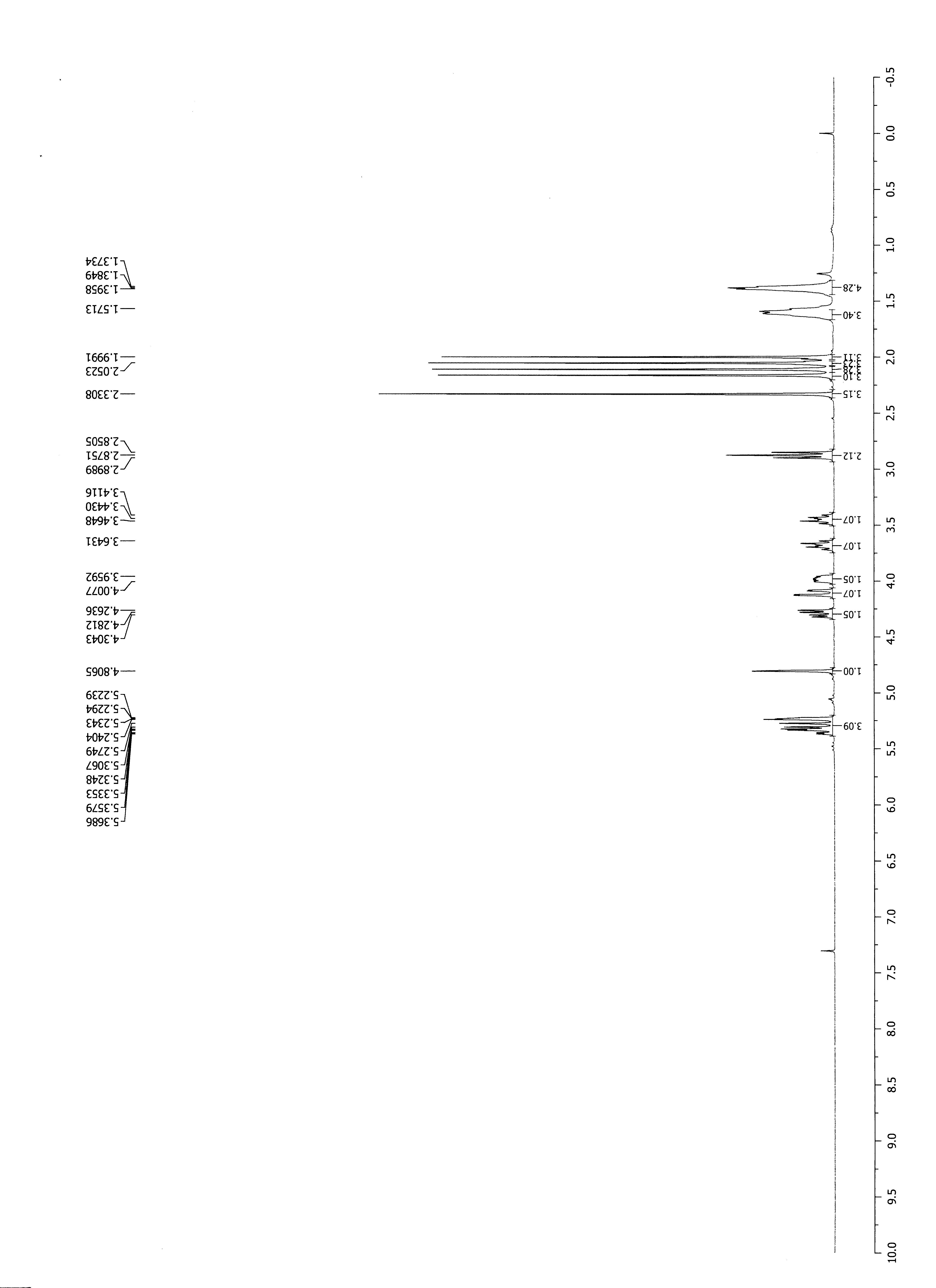
*

^13^C NMR spectrum of 6-thioacetylhexyl 2,3,4,6-tetra-*O*-acetyl-α-D-mannopyranoside **1b**

*
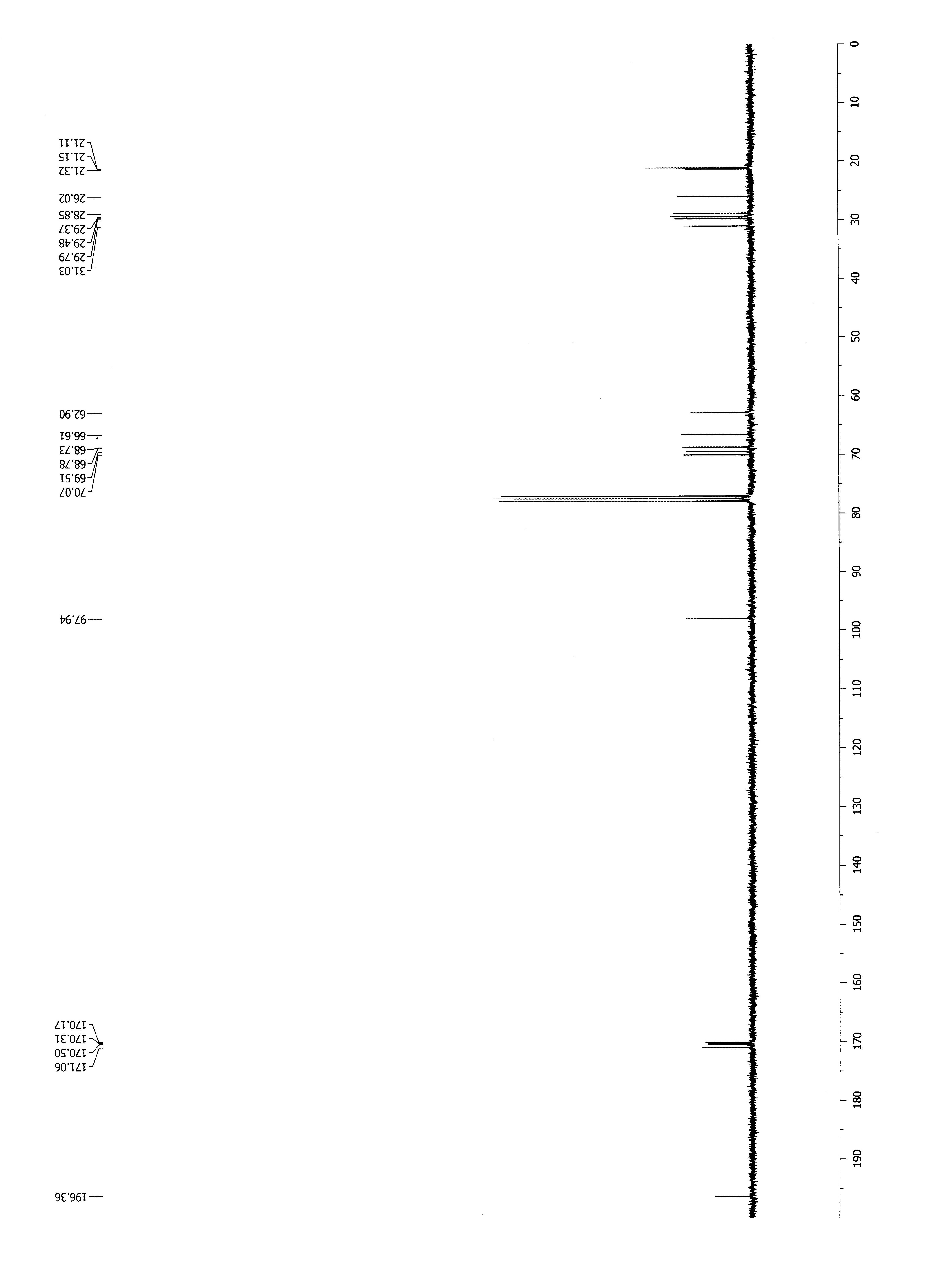
*

^1^H NMR spectrum of 6-mercaptohexyl α-D-mannopyranoside **1**

*
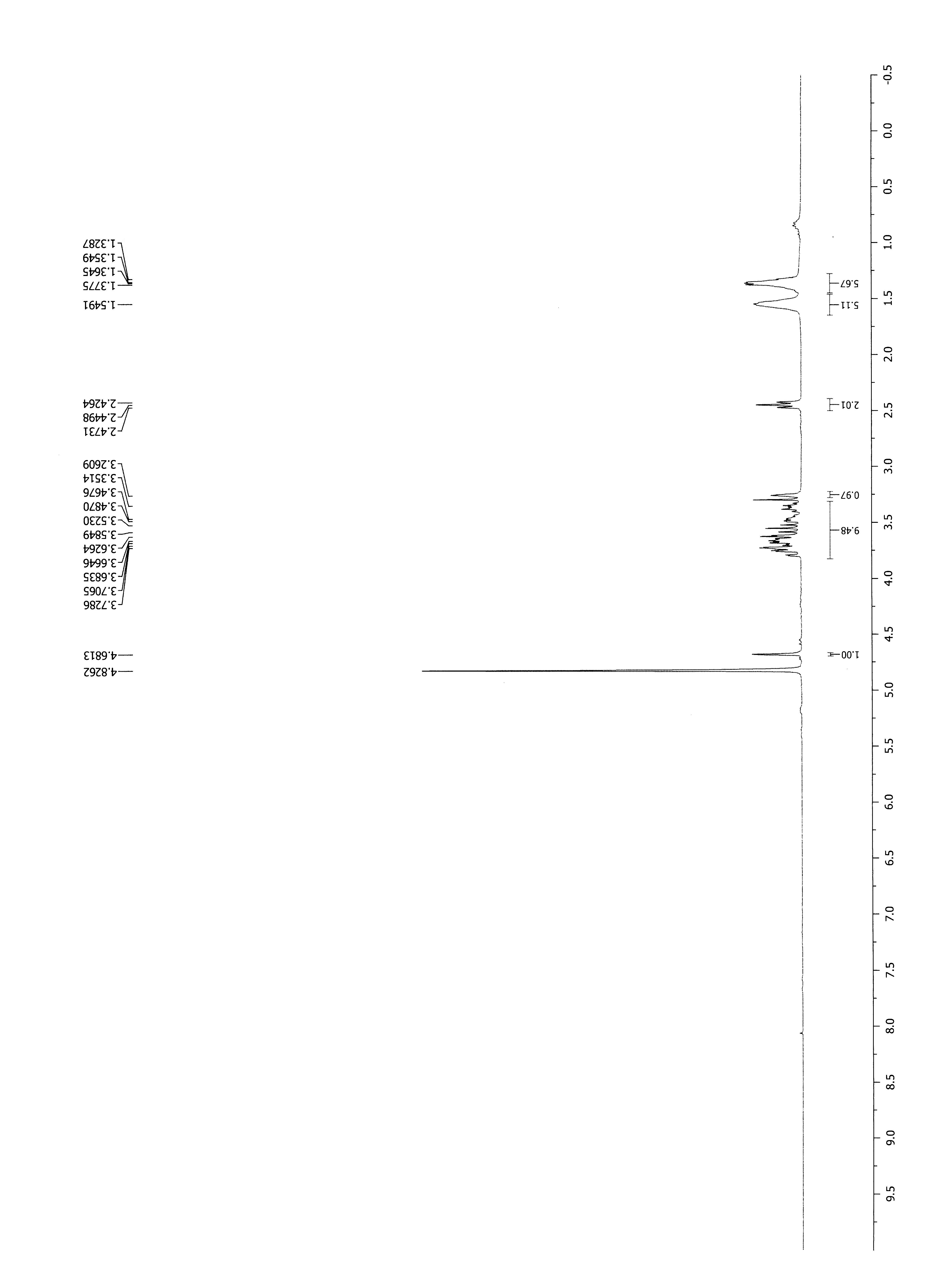
*

^13^C NMR spectrum of 6-mercaptohexyl α-D-mannopyranoside **1**

*
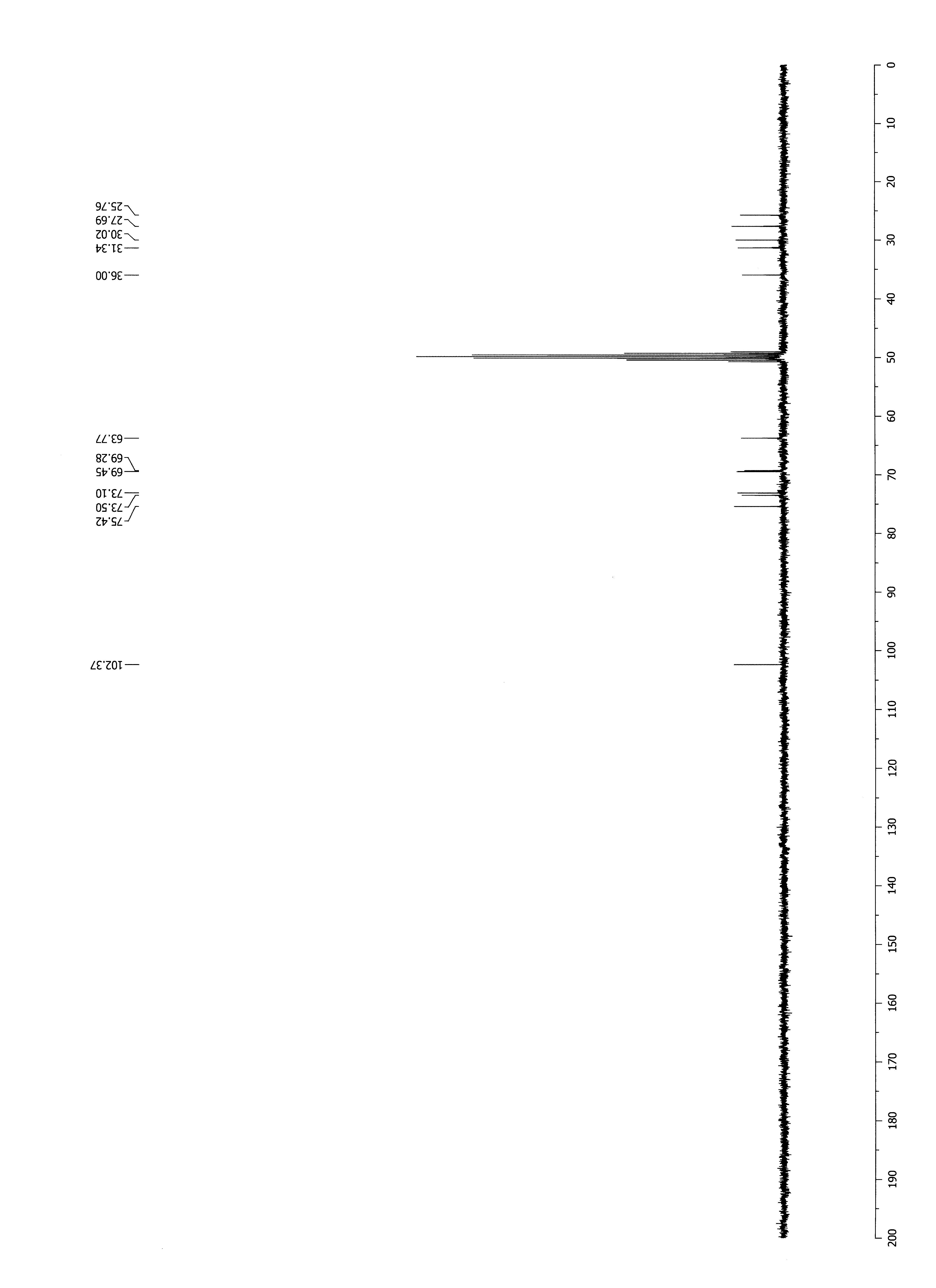
*

**Additional Figures**
